# What Causes Aplastic Anaemia: Results of Transplants from Genetically-Identical Twins

**DOI:** 10.21203/rs.3.rs-2540187/v1

**Published:** 2023-02-03

**Authors:** Robert Gale, Wolfgang Hinterberger, Neal S. Young, Andrew GENNERY, Christopher Dvorak, Kyle Hebert, Michael Heim, Larisa Broglie, Mary Eapen

**Affiliations:** Imperial College London; Danube Hospital, Donauspital, Klinik Donaustadt; National Heart, Lung, and Blood Institute, NIH; Newcastle University; UCSF; CIBMTR Statistical Center, Medical College of Wisconsin; CIBMTR^®^ (Center for International Blood and Marrow Transplant Research), Medical College of Wisconsin; CIBMTR^®^ (Center for International Blood and Marrow Transplant Research), Medical College of Wisconsin; Medical College of Wisconsin

**Keywords:** Aplastic anaemia, haematopoietic cell transplant, bone marrow failure, immune aetiology, bone marrow micro-environment

## Abstract

**Background:**

Aplastic anaemia has diverse aetiologies. Distinguishing between these is, in part, testable by analyzing results of haematopoietic cells transplants between genetically-identical twins one of whom has aplastic anaemia.

**Objective:**

Describe outcomes of genetically-identical twin transplants for aplastic anaemia with and without pretransplant conditioning.

**Methods:**

We interrogated data from an observational database of 59 consecutive recipients of genetically-identical twin transplants for aplastic anaemia reported to the Center for International Blood and Marrow Transplant Research (CIBMTR) 2000–2019.

**Results:**

38 subjects were male. Median age was 18 years (Interquartile Range [IQR], 11–32 years). Median interval from diagnosis to transplant was 2 months (IQR 1–3 months). 11 subjects received a 1^st^ transplant without pretransplant conditioning. 2 of recovered normal bone marrow function. The other 9 received a 2^nd^ transplant with pretransplant conditioning 7 of whom recovered. 48 subjects received pretransplant conditioning before a 1^st^ or 2^nd^ transplant all of whom recovered.

**Conclusion:**

Only some genetically-identical twins with aplastic anaemia recover normal bone marrow function after a 1^st^ haematopoietic cell transplant without pretransplant conditioning whereas most subjects recover when a transplant is preceded by pretransplant conditioning. These data are consistent with an immune-mediated aetiology of aplastic anaemia in most cases.

## Introduction

Aplastic anaemia has diverse etiologies including; (1) direct damage to haematopoietic stem or progenitor cells such as from chemicals, drugs and ionizing radiations; (2) an abnormal bone marrow micro-environment, (3) immune-mediated mechanism(s); or (4) combination of these. Aetiologies may differ between persons with similar phenotypes and even genotypes^1^. These aetiologies are not mutually-exclusive and can be tested, in part, by analyzing results of haematopoietic cell transplants between genetically-identical twins one of whom has aplastic anaemia. Recovery of bone marrow function after transplanting haematopoietic cells from a twin without pretransplant conditioning favours damage to haematopoietic stem or progenitor cells as the aetiology and suggests a bone marrow micro-environment abnormality is an unlikely aetiology. It also likely excludes a congenital abnormality as aetiology because the donor twin has normal bone marrow function. In contrast, failure to recover after a 1st unsuccessful transplant without pretransplant conditioning, but recovery after a 2nd transplant with it sometimes combined with posttransplant immune suppression, favours an immune aetiology.

There are several reports of genetically-identical twin transplants for aplastic anaemia, mostly of few subjects or with contradictory outcomes^2,3^. A prior Center for International Blood and Marrow Transplant Research (CIBMTR) study analyzed data from 40 subjects with aplastic anaemia receiving genetically-identical twin transplants from 1964–1992 with and without pretransplant conditioning^4^. About one-third of recipients of a twin transplant without pretransplant conditioning recovered normal bone marrow function; the remainder did not. We re-analyzed this question in a recent CIBMTR dataset 2000–2019 of 59 consecutive subjects. We found bone marrow recovery uncommon after a 1st transplant without pretransplant conditioning suggesting damage to haematopoietic stem or progenitor cells is an uncommon aetiology of bone marrow failure in persons with aplastic anaemia^2,5–10^.

## Methods And Materials

### Data Source

The Center for International Blood and Marrow Transplant Research (CIBMTR) is a research collaboration between the National Marrow Donor Program (NMDP)/Be The Match and the Medical College of Wisconsin (MCW). More than 330 medical centers worldwide submit clinical data to the CIBMTR about haematopoietic cell transplantation and other cell therapies. The CIBMTR Research Database includes long-term clinical data of more than 600,000 subjects. The CIBMTR collects subject and transplant co-variates using Transplant Essential Data forms on all subjects. Data are collected pretransplant, 100 days and 6 months posttransplant, annually until year 6 posttransplant and biannually thereafter. Subjects are followed longitudinally until death or loss to follow-up. Accuracy of reported data and compliance are monitored by on-site audits. Consent is sought from subjects and/or their legal guardians for research. The Institutional Review Board of the National Marrow Donor Program approved the study.

### Subjects

We interrogated data from 59 consecutive subjects with aplastic anaemia receiving a 1st haematopoietic cell transplants from a genetically-identical twin between January 1, 2000 to December 31, 2019. Subjects received bone marrow or blood cell graft.

### Outcomes and Definitions

We refer to giving haematopoietic cells intravenously between genetically-identical twins as a hematopoietic *transplant* but it can also be considered an *infusion*. The outcome of interest was bone marrow recovery defined as a granulocyte concentration > 0.5 × 10 E + 9/L and a platelet concentration > 20 ×10 10E + 9/L for 3 consecutive days. In some instances there was recovery to a normal blood granulocyte concentration but incomplete recovery to a normal platelet concentration. Analyses are descriptive.

## Results

We studied 59 consecutive subjects whose subject-, disease- and transplant-related co-variates are displayed in Table 1. Subjects were divided into those in whom a 1st transplant was preceded (N = 11) or not (N = 48) by pretransplant conditioning and posttransplant immune suppression. 38 subjects were male. Median age was 18 years (IQR 11–32 years). Median interval from diagnosis to 1st transplant was 2 months (IQR 1–3 months). 33 subjects received no immune suppression in the interval before starting their transplant whilst 9 failed prior immune suppression therapy mostly with cyclosporine and anti-thymocyte globulin (ATG) with or without corticosteroids. Pretransplant conditioning for a 1st transplant was predominately cyclophosphamide (N = 45) with (N = 33) or without other drugs including ATG (N = 19), fludarabine (N = 5), fludarabine plus ATG (N = 3), busulfan (N = 3) or ionizing radiation (2–10 Gy; N = 3). 1st grafts were bone marrow (N = 33) or mobilized blood cells (N = 26). 27 recipients of a 1st transplant with pretransplant conditioning received posttransplant immune suppression with a calcineurin-inhibitor alone (N = 14) or with methotrexate (N = 12) or mycophenolate mofetil (N = 1).

9 of 11 subjects of a 1st transplant without pretransplant conditioning had data reported on granulocyte and platelet recovery – 6 initially recovered both, 2 recovered granulocytes but not platelets and 1, neither. Only 2 recipients had a sustained granulocyte concentration > 0.5 × 10E + 9/L. The 9 subjects whose first infusion failed to restore normal bone marrow function received a 2nd transplant using the same twin donor with pretransplant conditioning. 7 recovered the target granulocyte and platelet concentrations. 2 subjects who did not recover after their 2nd transplant, 1 with early and 1 with late declines in concentrations received cyclophosphamide and ATG without additional posttransplant immune suppression as preparation for their 2nd infusion (Table 2).

In subjects receiving pretransplant conditioning for their 1st transplant, 46 had data reported on granulocyte recovery and 41 on platelet recovery. All had initial granulocyte recovery and 41, initial platelet recovery. However, granulocyte declines occurred in 7 subjects all of whom subsequently recovered normal bone marrow function after receiving transplants from an HLA-matched sibling donor (N = 3) or the same identical twin donor.

Graft-*versus* host disease (G*v*HD) was reported in 7 subjects. Neither acute nor chronic G*v*HD was reported in the 11 subjects transplanted without pretransplant conditioning. subjects receiving pre-transplant conditioning were reported to have acute GvHD ≥ grade-2 and 4 were reported to have chronic G*v*HD.

All subjects transplanted without pretransplant conditioning survived at a median follow-up of 6.5 years (range, 1–18 years). 45 of 48 subjects transplanted after receiving pretransplant conditioning survived at a median follow-up of 6 years (range, 0.5–16 years).

## Discussion

Our data indicate few recipients of a haematopoietic cell transplant from a genetically-identical twin without pretransplant conditioning recover normal blood granulocyte concentrations. In contrast, most recover when a 2nd transplant is preceded by pretransplant conditioning. Genetically-identical twins whose 1st transplant was preceded by conditioning had more frequent recovery of bone marrow function. These data suggest direct damage to haematopoietic stem or progenitor cells is an unlikely aetiology of most cases of aplastic anaemia. Because most subjects failing their 1st transplant without pretransplant conditioning recovered after a 2nd transplant with pretransplant conditioning, an abnormal bone marrow micro-environment also seems an unlikely aetiology but cannot be excluded as pretransplant conditioning could potentially alter the bone marrow micro-environment.

Several subjects had complete granulocyte recovery but only partial platelet recovery. Because the lifespan of granulocytes is much briefer than platelets, granulocyte recovery is a better surrogate for recovery of bone marrow function. Additionally, some recipients of successful haematopoietic cells allotransplants have impaired platelet recovery despite otherwise normal bone marrow function and complete haematopoietic chimerism. Diverse reasons for impaired platelet recovery are reviewed elsewhere^11–13^.

Although the data presented here seemingly differ from our prior study where 7 of 23 subjects recovered bone marrow function without pretransplant conditioning versus 2 of 11, this difference is not statistically significant (*P* = 0.73 with Yates correction)^4^.

There were several unexpected findings in our study. 1st, several subjects were reported to develop acute and/or chronic G*v*HD. Whether recipients of a genetically-identical twin transplant can develop G*v*HD is controversial^14^. 2nd, many subjects received posttransplant immune suppression, presumably to prevent G*v*HD despite transplants from presumed genetically-identical twins. Whether donors were really genetically-identical twins cannot be known with complete certainty as genetic-identity of twins is a probabilistic estimate. Additionally, the CIBMTR relies on centres to report presence of G*v*HD and this can be subjective and confounded by heuristics^15–22^. Incorrect diagnosis of chronic G*v*HD seems far less likely but a syndrome resembling chronic GvHD is reported in rats receiving syngeneic grafts in the context of posttransplant cyclosporine^23^.

There are important limitations to our study that are related to the use of an observational database. 1st, there are relatively few subjects. However, our cohort likely represents the majority of genetically-identical twin transplants for aplastic anaemia performed in the US. 2nd, as indicated, we rely on reporting from centres to identify the donor as a genetically-identical sibling and to have normal haematopoiesis. No central testing was done to confirm the status of the donor and recipient beyond routine HLA-typing and additional genetic differences may be present that is not able to be tested using current methods. 3rd, when there is recovery of bone marrow function after pretransplant therapy and infusion of twin haematopoietic cells it is impossible to determine whether this recover was endogenous of from twin cells unless the graft is genetically marked. This was not done in any of the transplants we studied. Lastly, we do not know why some subjects received pretransplant conditioning before a 1st transplant and others did not which may have resulted in a selection biases. Similarly, we do not know why some reportedly genetically-identical twins received posttransplant immune suppression. Because graft-rejection cannot occur between genetically-identical persons preventing this cannot be the reason. In addition, the rationale for why some subjects received immune suppression before attempting a transplant and others did not is unknown. And we do not know what proportion of persons with a genetically-identical twin recovered after receiving other interventions such as immune suppression and are not included in our dataset. Given these limitations one should not use results of our analyses to define a preferred transplant strategy for persons with aplastic anaemia with a potential genetically-identical twin donor.

In conclusion, our data indicate transplanting presumably normal haematopoietic cells from a likely genetically-identical twin without pretransplant conditioning rarely results in recovery of bone marrow function. These data are consistent with the hypothesis that relatively few cases of aplastic anaemia are caused by direct damaged to haematopoietic stem or progenitor cells. The data are also consistent with the hypothesis that an immune-mediated aetiology underlies most cases of aplastic anaemia although bone marrow micro-environment abnormalities cannot be excluded.

## Figures and Tables

**Table 1:** Patient and transplant characteristics of patients undergoing first HSCT for aplastic anemia with identical twin donor, 2000–2019 (N; %)

	No conditioning	Conditioning
N Subjects	11	48
Age at transplant, years		
Median (min-max)	20 (2–71)	18 (1–70)
< 1–17	4 (36)	24 (50)
18–39	5 (45)	17 (35)
40+	2 (18)	7 (15)
Sex		
Male	6 (55)	32 (67)
Female	5 (45)	16 (33)
Karnofsky performance score		
80–100	7 (64)	39 (81)
< 80	0 (0)	2 (4)
Not reported	4 (36)	7 (15)
Interval from diagnosis to transplant, mo		
< 4	11 (100)	37 (77)
4 to 12	0 (0)	8 (17)
> 12	0 (0)	3 (6)
Graft-type		
Bone marrow	3 (27)	30 (63)
Blood	8 (73)	18 (38)
Conditioning regimen		
None	11 (100)	0 (0)
Cy/ATG	0 (0)	19 (40)
Cy/Flu/ATG	0 (0)	3 (6)
Cy/Flu	0 (0)	5 (10)
Cy alone	0 (0)	12 (25)
Flu/ATG	0 (0)	3 (6)
Bu/Cy	0 (0)	3 (6)
TBI 2 Gy/Cy/Flu	0 (0)	1 (2)
TBI 10 Gy/Cy	0 (0)	2 (4)
GVHD prophylaxis		
None given	10 (91)	21 (44)
CNI + MTX	0 (0)	12 (25)
CNI + MMF	1 (9)	1 (2)
CNI alone	0 (0)	14 (29)
Transplant year		
2000–2004	5 (45)	9 (19)
2005–2009	2 (18)	13 (27)
2010–2014	4 (36)	17 (35)
2015–2019	0 (0)	9 (19)
Follow-up, mo - median (range)	78 (12–216)	73 (6–195)

**Table 2:** Characteristics and outcomes following subsequent hematopoietic stem cell transplant, for applicable cases

Conditioning for 1st transplant	Age at 2nd transplant (y)	Interval between transplants (mo)	Donor	Graft-type	Conditioning for 2nd transplant	*GvHD* prophylaxis	Year of 2nd transplant	Outcome	Follow-up from 2nd transplant (yr)	Granulocyte recovery	Platelet recovery
No	19	3	Twin	Blood	Cy/ATG	CNI + MTX	2001	Alive	9.9	Yes	Yes
No	17	7	Twin	Blood	Cy	None	2002	Alive	0.4	Yes	Yes
No	47	5	Twin	Blood	Cy/ATG	CNI + MMF	2003	Alive	13	Yes	Yes
No	34	1	Twin	Blood	Cy alone	None	2003	Alive	3.25	Yes	Yes
No	2	1	Twin	Blood	Cy/ATG	None	2006	Alive	14.5	No	No
No	21	10	Twin	Blood	TBI/Cy	None	2008	Alive	8.67	Yes	Yes
No	35	8	Twin	Blood	Cy/ATG	None	2012	Alive	5.75	Yes	Yes
No	5	3	Twin	BM	Cy/ATG	None	2013	Alive	6.16	Yes	Yes
No	24	4	Twin	Blood	Cy	None	2015	Alive	3.58	Yes	Yes
Cy/ATG	22	55	Twin	Blood	Cy/ATG	None	2008	Alive	10	Yes	Yes
Cy/ATG	7	12	MSD	BM	Cy/Flu/ATG	None	2012	Alive	5	Yes	Yes
Cy/ATG	7	42	Twin	BM	Cy/Flu/ATG	None	2013	Alive	3.25	Yes	Yes
Cy/ATG	20	51	Twin	Blood	Cy	None	2017	Alive	3	Yes	Yes
Cy	33	50	Twin	Blood	Bu/Cy	CNI + MTX	2010	Alive	2.75	Yes	Yes
Cy/Flu/ATG	15	5	MSD	Blood	ATG	None	2011	Alive	8.33	Yes	Yes
Cy/Flu	47	27	MSD	Blood	Flud	CNI + MTX	2018	Alive	2.1	Yes	Yes

Abbreviations: Cy, Cyclophosphamide; ATG, anti-thymocyte globulin; Flu, fludarabine; MSD, HLA-matched sibling donor;BM; bone marrow; Bu, busulfan; CNI, C mycophenolate mofetil; GvHD, graft-*versus*-host disease.
